# Comparative discriminatory performance of emerging endocrine-metabolic indices versus obesity indices for cardiometabolic multimorbidity in older adults: a cross-sectional study

**DOI:** 10.3389/fendo.2026.1776998

**Published:** 2026-05-20

**Authors:** Haosen Pan, Qianqian Zhang, Yuanyuan Wang, Ankai Chen, Xintao Chen, Liping Ren, Maoyun Miao

**Affiliations:** 1 School of Nursing, Shandong Second Medical University, Weifang, China; 2 Xinhua Hospital, Weifang, China

**Keywords:** aged, cardiometabolic multimorbidity, endocrine indices, metabolic indices, obesity indices

## Abstract

**Study objectives:**

The objective of this study was to assess and compare the discriminatory performance of endocrine-metabolic and obesity indices for identifying cardiometabolic multimorbidity (CMM) in older adults.

**Methods:**

A total of 4,173 elderly people who participated in community physical examinations in a community hospital in Shandong Province, China in 2024 were included in this study. Obesity indicators include Body Roundness Index (BRI), Chinese Visceral Adiposity Index (CVAI), A Body Shape Index (ABSI) and Relative Fat Mass (RFM); Endocrine-metabolic indices include the Triglyceride/High-Density-Cholesterol (TG/HDL-C) ratio, Metabolic Score for Insulin Resistance (METS-IR), Triglyceride-Glucose (TyG) index and Atherogenic Index of Plasma (AIP). Multivariable logistic regression was used to examine associations with CMM, with quartile analyses for dose-response trends. Restricted cubic spline models were applied to assess potential nonlinearity. Discriminatory performance was evaluated using receiver operating characteristic (ROC) curves and area under the curves (AUCs), and AUCs were compared. Subgroup and sensitivity analyses were conducted to assess robustness.

**Results:**

In the fully adjusted model, all eight indices were all independently positively correlated with the risk of CMM, with significant dose-response trends across quartiles (all P for trend< 0.05). METS-IR showed the strongest risk gradient, with an OR of 6.79 (95% CI 4.85-9.52) comparing the fourth versus the first quartile. Restricted cubic spline analyses suggested largely linear associations for most indices, whereas TG/HDL-C and METS-IR exhibited significant nonlinear relationships, with steeper increases in CMM risk at higher levels. In ROC analyses, endocrine-metabolic indices generally demonstrated higher discriminatory performance than obesity indices. METS-IR yielded the highest adjusted AUC (0.685), followed by TyG (AUC 0.664). Among obesity indices, CVAI showed comparatively better performance (AUC 0.646). Subgroup and sensitivity analyses yielded consistent results.

**Conclusions:**

In this community-based sample of older adults, endocrine-metabolic indices showed relatively stronger associations and generally better discriminatory performance for CMM than obesity indices. METS-IR demonstrated the most favorable overall performance and may be a practical indicator for identifying older individuals at increased risk of CMM in routine community health examinations.

## Introduction

Cardiometabolic multimorbidity (CMM) is generally defined as an individual having two or more cardiovascular metabolic diseases (CMDs), such as hypertension, diabetes, coronary artery disease, or stroke (1). As the global population ages, the coexistence of multiple diseases has become a significant burden on chronic disease prevention and control, and its prevalence continues to rise worldwide (2). Previous studies have shown a significant positive correlation between the prevalence of CMM and population aging (3). Epidemiological data shows that the global prevalence of CMM is approximately 8.8%, with the highest prevalence in North America at 14.4% (4). In China, the prevalence of CMM among community dwelling older adults has been reported to reach 25.3% (5). It is worth noting that the risk of all-cause mortality increases in a gradient with the number of comorbidities (6). Compared with people without CMD, those who develop CMM can have an approximately three times higher risk of all-cause mortality (7). Individuals with CMM may lose up to 15 years of life expectancy at age 60, which is approximately twice the reduction observed among those with a single CMD (8). Multimorbidity has become a critical concern in the global management and care of chronic diseases in the elderly (9). Therefore, assessing the predictive value of different indicators for CMM in the elderly population is of great significance, as it can help achieve early identification and precise care intervention for at-risk populations, thereby reducing the risk of disease occurrence and death.

In recent years, studies have consistently shown that endocrine metabolic dysregulation and excessive visceral fat accumulation play key roles in the development and clustering of cardiometabolic disorders, often accompanied by adverse lipid profiles and insulin resistance (IR) related phenotypes (10, 11). However, direct assessment of visceral adiposity and metabolic disturbances in routine practice may be constrained by practical barriers, including time consumption, higher costs, and limited feasibility of certain procedures in large scale screening, particularly in older populations (12, 13). Against this background, researchers have proposed a range of simple, economical, and accessible surrogate indices derived from routine anthropometric measures or blood biochemical parameters to support screening and risk stratification for CMM (14).

Among anthropometric based indicators, several novel obesity indices have been developed to better capture adiposity level and fat distribution patterns beyond conventional measures (15–18). Thomas et al. (2013) proposed the concept of the Body Roundness Index (BRI), which uses the geometric relationship between waist circumference (WC) and height to quantify torso roundness and indirectly reflect abdominal fat accumulation (16). The Chinese Visceral Adiposity Index (CVAI) was developed by Xia et al. based on visceral adiposity index framework, integrating age, body mass index (BMI), WC, triglycerides (TG), and high-density lipoprotein cholesterol (HDL-C) as a clinical index tailored to the Chinese population and used as a surrogate marker of visceral fat accumulation (17). The A Body Shape Index (ABSI) is derived from the residual of WC after adjustment for height and BMI, aiming to reduce body size related confounding and highlight abdominal obesity characteristics (15). Relative Fat Mass (RFM), which uses the WC-to-height ratio as its core metric and adjusts for sex, provides a simple tool to estimate relative body fat level and overall adiposity (18).

In parallel, another group of emerging indices focuses more directly on endocrine-metabolic related abnormalities using routine blood biochemical parameters (19–22). The Triglyceride/High-Density-Cholesterol (TG/HDL-C) ratio is a simple phenotypic indicator of lipid imbalance and has been used to indicate decreased insulin sensitivity and related endocrine abnormalities (19). Metabolic Score for Insulin Resistance (METS-IR) integrates fasting plasma glucose (FPG), TG, HDL-C, and BMI to estimate the level of IR without direct insulin measurement, thereby reflecting the degree of endocrine regulatory imbalance (21). The Triglyceride-Glucose (TyG) index is calculated from the logarithmic product of TG and FPG, and it is widely regarded as one of the surrogate indicators of IR because of its accessibility and stability (20). The Atherogenic Index of Plasma (AIP), a logarithmic transformation of TG/HDL-C, summarizes atherogenic lipid characteristics in a concentrated manner (22).

Previous studies have reported significant associations of BRI, CVAI, ABSI, RFM, TG/HDL-C, METS-IR, TyG, and AIP with CMM in the general population (23–28). However, much of the existing evidence has focused on general populations and has typically evaluated indices from a single pathway in isolation, despite the central roles of lipid dysregulation and insulin-resistance-related phenotypes in cardiometabolic clustering (23, 29, 30). Consequently, head-to-head comparisons of endocrine-metabolic indices versus obesity indices within the same older-adult population remain limited (31), and the relative discriminatory utility of these two index families for identifying CMM is still unclear. To address this gap, the present cross-sectional study simultaneously examined four endocrine-metabolic indices and four obesity indices in older adults and compared their discriminatory performance for CMM, aiming to identify a practical index for CMM screening in older adults.

## Methods

### Study population

The data for this study came from elderly residents in the community who underwent health checkups at a community hospital in Shandong Province in 2024. The study protocol was approved by the Shandong Second Medical University Ethics Committee (Approval No.: SDSMU-2025-YX-201). This study followed the ethical standards of the Declaration of Helsinki, and all participants signed informed consent forms.

**Figure 1:**
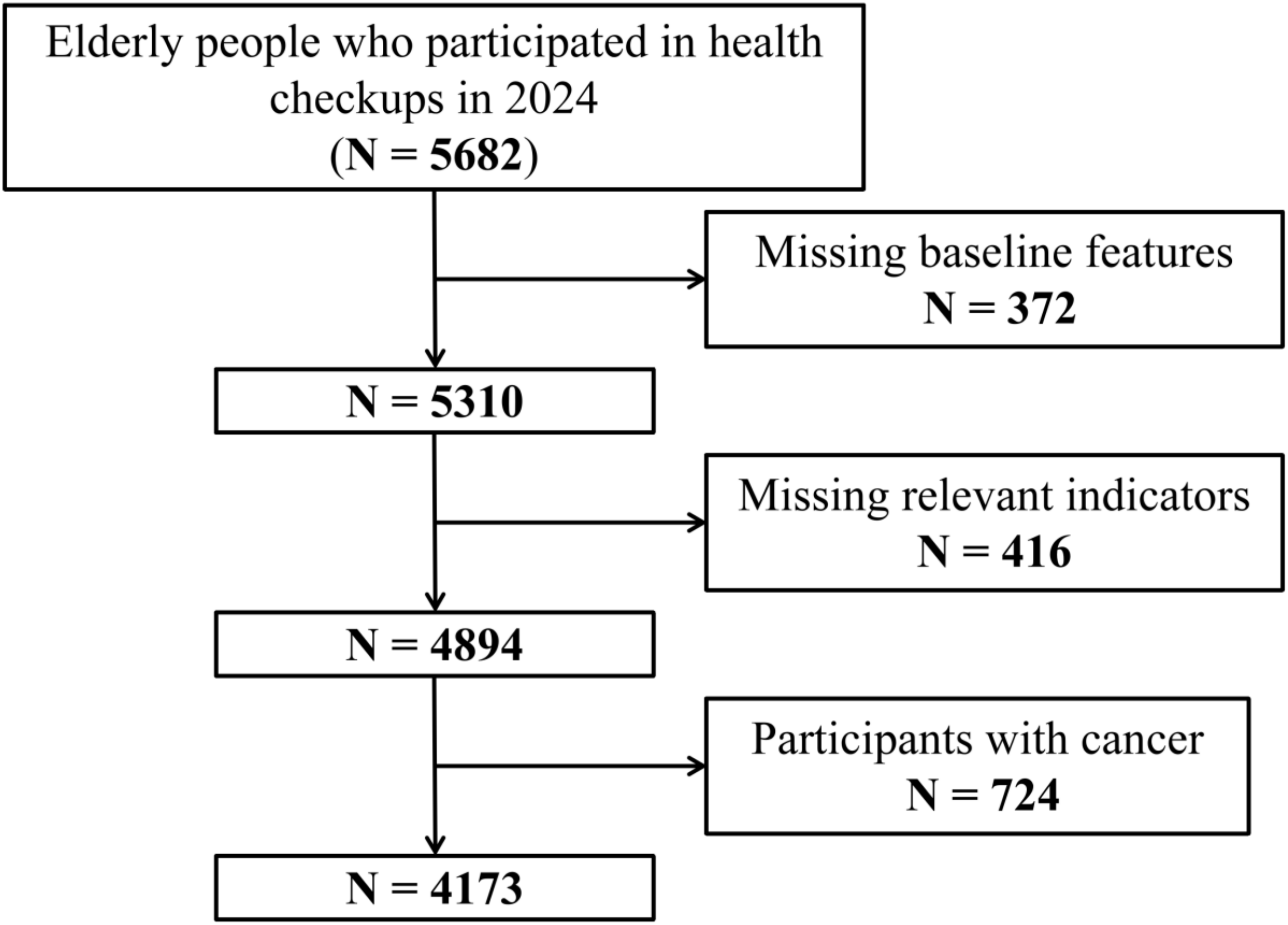
Research flow chart.

In this study, our inclusion criteria were elderly residents (≥60 years old) in the community who completed health checkups in 2024. Of the 5682 individuals assessed, we excluded participants with missing baseline characteristics (n=372), missing relevant indicators (n=416), and those with cancer (n=721). Ultimately, our study included 4173 participants with complete and reliable information, representing an inclusion rate of 73.44% (**Figure 1**).

### Data collection

In this study, baseline data were obtained through structured questionnaires, physical examinations, and laboratory tests, all conducted by professionals. Demographic and lifestyle variables included age, sex, alcohol consumption, smoking status, and exercise frequency. Physical examinations included height, weight, blood pressure, heart rate (HR), and WC. BMI was calculated by dividing weight (kg) by the square of height (m). Self-reported physician diagnoses of diabetes, hypertension, stroke, and heart disease were recorded. Laboratory tests included FPG, serum alanine aminotransferase (ALT), serum aspartate aminotransferase (AST), total bilirubin (TBIL), serum creatinine (SCr), blood urea nitrogen (BUN), total cholesterol (TC), TG, low-density lipoprotein cholesterol (LDL-C), and HDL-C. Fasting venous blood samples were collected and analyzed by trained medical personnel from the Chinese Center for Disease Control and Prevention according to standardized procedures. Disease definitions followed standards (32, 33). Diabetes mellitus: Fasting blood glucose ≥ 126 mg/dL, currently taking hypoglycemic medication, or the patient reports a doctor’s diagnosis of diabetes. Hypertension: Systolic blood pressure (SBP) ≥ 140 mmHg, diastolic blood pressure (DBP) ≥ 90 mmHg, currently taking antihypertensive medication, or the patient reports a doctor’s diagnosis of hypertension.

### Definition of adiposity indicators

In this study, BRI (16), CVAI (17), ABSI (15), and RFM (18) were calculated based on anthropometric parameters. The formulas (Equations 1–4) are as follows:

(1) $$\text{BRI}=364.2-365.5\times \sqrt{1-{\left(\frac{\text{wc}}{2\pi }\right)}^{2}/{\left(\frac{\text{Height}}{2}\right)}^{2}}$$

(2) $$\begin{array}{l} \text{Male: CVAI}=-267.93+0.68\times \text{Age}+0.03\times \text{BMI}+4.00\times \text{WC}+22.00\times \log_{10}(\text{TG})-16.32\times \text{HDL-C};\\ \text{Female: CVAI}=-187.32+1.71\times \text{Age}+4.23\times \text{BMI}+1.12\times \text{WC}+39.76\times \log_{10}(\text{TG})-11.66\times \text{HDL-C} \end{array}$$

(3) $$\text{ABSI}=\frac{\text{WC}}{{\text{BMI}}^{2/3}\times {\text{Height}}^{1/2}}$$

(4) $$\begin{array}{l} \text{Male: RFM}=64-20\times \frac{\text{Height}}{\text{WC}};\\ \text{Female: RFM}=76-20\times \frac{\text{Height}}{\text{WC}} \end{array}$$

### Definition of endocrine-metabolic indicators

From relevant clinical and biochemical parameters, we indirectly derived four endocrine-metabolic indices—TG/HDL-C (19), METS-IR (21), TyG (20), and AIP (22). The formulas (Equations 5–8) are as follows:

(5) $$\text{TG}/\text{HDL}-\text{C ratio}=\frac{\text{TG}}{\text{HDL-C}}$$

(6) $$\text{METS-IR}=\frac{\ln(2\times \text{FPG}+\text{TG})\times \text{BMI}}{\ln(\text{HDL-C})}$$

(7) $$\text{TyG}=\ln(\frac{\text{TG}\times \text{FPG}}{2})$$

(8) $$\text{AIP}=\log_{10}(\frac{\text{TG}}{\text{HDL-C}})$$

### Definition of CMM

This study collected participants’ past medical history and self-reported physician diagnoses through questionnaires. CMM was defined as the coexistence of two or more CMDs. In this study, CMDs included hypertension, diabetes, heart disease, and stroke; if at least two of these diseases coexisted, it was defined as cardiovascular metabolic comorbidity (34).

### Statistical analysis

Participants were categorized according to their CMM status. Normally distributed continuous variables are expressed as mean ± standard deviation (SD), non-normally distributed continuous variables are expressed as median and interquartile range (IQR), categorical variables are expressed as frequency and percentage (%), and differences between groups are assessed using the chi-square test; differences in continuous variables are assessed using the t-test or Wilcoxon rank-sum test. Considering that the relevant indicators may be skewed in the general population and that extreme values may have potential clinical significance, this study did not subjectively remove outliers, and all observations were included in the analysis.

We included BRI, CVAI, ABSI, RFM, TG/HDL-C, METS-IR, TyG, and AIP in both continuous variable and quartile stratification (Q1-Q4) to explore their association with CMM. We constructed multivariable logistic regression models to examine the associations of BRI, CVAI, ABSI, RFM, TG/HDL-C, METS-IR, TyG, and AIP with the risk of CMM, and expressed the results as odds ratios (ORs) with 95% confidence intervals (CIs). In our progressively adjusted multivariate model, we included Age, Gender, Smoking, Drinking, Exercise, SBP, DBP, HR, and BMI. Meanwhile, based on the multi-factor adjusted logistic regression model, we use the restricted cubic spline (RCS) method to examine the potential nonlinear relationship between each variable and the CMM. We performed receiver operating characteristic (ROC) analysis and calculated the area under the curve (AUC) to assess and compare the discriminative ability of obesity indicators (BRI, CVAI, ABSI, and RFM) and endocrine-metabolic indices (TG/HDL-C, METS-IR, TyG, and AIP) in identifying the risk of CMM.

We conducted stratified analyses by gender (male or female), SBP (<140 mmHg or ≥140 mmHg), BMI (<24 kg/m² or ≥24 kg/m²), exercising (never, occasionally, at least once a week, or daily), smoking (never, currently smoking, or quit smoking), and drinking (never, occasionally, frequently, or daily). To evaluate potential effect modification, we added interaction terms to the multivariable regression models. Finally, we conducted sensitivity analyses by additionally incorporating TC and LDL-C into the model to evaluate the robustness of the results.

R version 4.5.1 was used for all statistical computations, with two-sided P< 0.05 indicating statistical significance.

## Result

### Baseline characteristics of the study population

This study included 4173 participants, with 3012 in the non-CMM group and 1161 in the CMM group. Compared with the non-CMM group, the CMM group had higher levels of age, SBP, weight, BMI, HR, WC, FPG, SCr, BUN, TG, and eight other indicators (all p< 0.05). There were no significant differences between the two groups in terms of sex, DBP, height, ALT, and AST (all P > 0.05). The CMM group had a higher proportion of never-exercising individuals, a lower proportion of current smokers, but a higher proportion of former smokers and a lower proportion of daily drinkers. Baseline characteristics of the two groups are shown in **Table 1**.

**Table 1:** Baseline characteristics by CMM status.

Characteristic	Total (n=4173)	Non-CMM (n=3012)	CMM (n=1161)	P
Age	69.92 ± 5.63	69.56 ± 5.42	70.84 ± 6.06	<0.001^a^
Gender (%)				0.647^b^
Male	1774(42.5%)	1287(42.7%)	487(41.9%)	
Female	2399(57.5%)	1725(57.3%)	674(58.1%)	
SBP	139.09 ± 18.97	137.91 ± 18.87	142.14 ± 18.88	<0.001^a^
DBP	80.16 ± 10.95	80.26 ± 11.00	79.90 ± 10.82	0.331^a^
Height	161.19 ± 8.39	161.28 ± 8.33	160.97 ± 8.52	0.284^a^
Weight	66.51 ± 10.62	65.93 ± 10.55	68.00 ± 10.68	<0.001^a^
Exercising (%)				<0.001^b^
Never	529(12.7%)	342(11.4%)	187(16.1%)	
Occasionally	171(4.1%)	113(3.8%)	58(5%)	
At least once a week	149(3.6%)	114(3.8%)	35(3%)	
Daily	3324(79.7%)	2443(81.1%)	881(75.9%)	
Smoking (%)				0.002^b^
Never smoked	3760(90.1%)	2702(89.7%)	1058(91.1%)	
Currently smoking	304(7.3%)	241(8%)	63(5.4%)	
Quit smoking	109(2.6%)	69(2.3%)	40(3.4%)	
Drinking (%)				0.029^b^
Never	3620(86.7%)	2586(85.9%)	1034(89.1%)	
Occasionally	223(5.3%)	166(5.5%)	57(4.9%)	
Frequently	58(1.4%)	44(1.5%)	14(1.2%)	
Daily	272(6.5%)	216(7.2%)	56(4.8%)	
BMI	25.55 ± 3.34	25.31 ± 3.33	26.19 ± 3.30	<0.001^a^
HR	69.16 ± 11.13	68.72 ± 10.93	70.29 ± 11.55	<0.001^a^
WC	88.08 ± 8.86	87.36 ± 8.83	89.97 ± 8.66	<0.001^a^
FPG	6.52 ± 1.79	6.16 ± 1.36	7.48 ± 2.35	<0.001^a^
ALT	19.18 ± 17.72	18.98 ± 19.55	19.68 ± 11.68	0.257^a^
AST	21.74 ± 16.64	21.87 ± 18.77	21.4 ± 9.00	0.420^a^
TBIL	14.13 ± 6.03	14.36 ± 6.28	13.52 ± 5.30	<0.001^a^
SCr	68.48 ± 23.84	67.68 ± 20.52	70.54 ± 30.74	<0.001^a^
BUN	5.49 ± 1.67	5.40 ± 1.44	5.71 ± 2.15	<0.001^a^
TC	5.07 ± 1.11	5.22 ± 1.05	4.66 ± 1.15	<0.001^a^
TG	1.55 ± 0.95	1.52 ± 0.92	1.63 ± 1.03	0.002^a^
LDL-C	2.89 ± 0.86	3.00 ± 0.82	2.61 ± 0.89	<0.001^a^
HDL-C	1.36 ± 0.34	1.39 ± 0.34	1.27 ± 0.31	<0.001^a^
BRI	4.35 ± 1.18	4.25 ± 1.16	4.61 ± 1.19	<0.001^a^
CVAI	125.04 ± 33.56	121.44 ± 33.67	134.35 ± 31.44	<0.001^a^
ABSI*1000	6.33 ± 0.43	6.31 ± 0.43	6.36 ± 0.43	<0.001^a^
RFM	33.95 ± 7.69	33.6 ± 7.72	34.88 ± 7.52	<0.001^a^
TG/HDL-C	1.97 ± 1.60	1.89 ± 1.50	2.21 ± 1.81	<0.001^a^
METS-IR	35.01 ± 6.01	34.24 ± 5.83	37.02 ± 5.99	<0.001^a^
TyG	8.83 ± 0.58	8.77 ± 0.55	9.00 ± 0.62	<0.001^a^
AIP	0.20 ± 0.28	0.18 ± 0.27	0.25 ± 0.28	<0.001^a^

^a^ P value from Wilcoxon rank sum test.

^b^ P value from Chi-square test.

### Associations and dose-response relationships between indices and CMM

In the logistic regression analyses (**Table 2**), all eight indices were positively associated with CMM (all P< 0.05). Although the effect sizes were attenuated in the fully adjusted model (Model 3), these indices remained independently associated with an increased risk of CMM, supporting their potential value as predictive biomarkers (all P< 0.05).

**Table 2:** Association of various indices with CMM in logistic regression models.

	n	Model 0		Model 1		Model 2		Model 3	
		OR (95% CI)	P	OR (95% CI)	P	OR (95% CI)	P	OR (95% CI)	P
BRI									
per SD change		1.29 (1.22, 1.37)	<0.001	1.28 (1.21, 1.36)	<0.001	1.28 (1.21, 1.36)	<0.001	1.15 (1.04, 1.26)	0.005
Q1	1049	1.00 (reference)		1.00 (reference)		1.00 (reference)		1.00 (reference)	
Q2	1039	1.41 (1.15, 1.73)	0.001	1.41 (1.15, 1.73)	0.0012	1.41 (1.15, 1.73)	0.001	1.24 (0.99, 1.55)	0.053
Q3	1042	1.65 (1.35, 2.02)	<0.001	1.63 (1.34, 1.98)	<0.001	1.64 (1.35, 1.99)	<0.001	1.29 (1.01, 1.65)	0.035
Q4	1043	2.24 (1.84, 2.73)	<0.001	2.18 (1.79, 2.65)	<0.001	2.19 (1.80, 2.67)	<0.001	1.48 (1.16, 1.89)	0.007
Ptrend			<0.001		<0.001		<0.001		0.011
CVAI									
per SD change		1.01 (1.01, 1.01)	<0.001	1.01 (1.01, 1.01)	<0.001	1.01 (1.01, 1.01)	<0.001	1.01 (1.01, 1.02)	<0.001
Q1	1045	1.00 (reference)		1.00 (reference)		1.00 (reference)		1.00 (reference)	
Q2	1043	1.56 (1.26, 1.93)	<0.001	1.54 (1.24, 1.91)	<0.001	1.55 (1.25, 1.92)	<0.001	1.52 (1.21, 1.91)	<0.001
Q3	1042	1.83 (1.49, 2.26)	<0.001	1.76 (1.43, 2.17)	<0.001	1.78 (1.45, 2.19)	<0.001	1.69 (1.31, 2.18)	<0.001
Q4	1043	2.93 (2.39, 3.58)	<0.001	2.71 (2.21, 3.32)	<0.001	2.71 (2.21, 3.32)	<0.001	2.48 (1.88, 3.27)	<0.001
Ptrend			<0.001		<0.001		<0.001		<0.001
ABSI									
per SD change		1.02 (1.01, 1.04)	<0.001	1.02 (1.00, 1.03)	0.025	1.02 (1.00, 1.03)	0.020	1.02 (1.01, 1.04)	0.001
Q1	1044	1.00 (reference)		1.00 (reference)		1.00 (reference)		1.00 (reference)	
Q2	1043	1.16 (0.95-1.41)	0.141	1.13 (0.93-1.38)	0.221	1.12 (0.92-1.37)	0.271	1.12 (0.91-1.37)	0.277
Q3	1043	1.43 (1.18-1.74)	<0.001	1.37 (1.13-1.67)	0.002	1.39 (1.14-1.69)	0.001	1.40 (1.15-1.71)	<0.001
Q4	1043	1.36 (1.12-1.65)	0.002	1.24 (1.02-1.52)	0.030	1.25 (1.03-1.53)	0.025	1.36 (1.11-1.67)	0.003
Ptrend			<0.001		0.008		0.005		<0.001
RFM									
per SD change		1.02 (1.01, 1.03)	<0.001	1.08 (1.06, 1.10)	<0.001	1.08 (1.06, 1.10)	<0.001	1.05 (1.02, 1.08)	0.002
Q1	1045	1.00 (reference)		1.00 (reference)		1.00 (reference)		1.00 (reference)	
Q2	1042	1.41 (1.16, 1.72)	<0.001	1.65 (1.34, 2.04)	<0.001	1.67 (1.35, 2.07)	<0.001	1.28 (1.01, 1.63)	0.041
Q3	1049	1.10 (0.90, 1.35)	0.329	2.08 (1.43, 2.99)	<0.001	2.12 (1.46, 3.08)	<0.001	1.38 (0.96, 1.99)	0.137
Q4	1037	1.72 (1.42, 2.09)	<0.001	3.13 (2.16, 4.54)	<0.001	3.18 (2.20, 4.59)	<0.001	1.60 (1.00, 2.57)	0.051
Ptrend			<0.001		<0.001		<0.001		0.04
TG/HDL-C									
per SD change		1.12 (1.08, 1.17)	<0.001	1.13 (1.09, 1.18)	<0.001	1.13 (1.08, 1.18)	<0.001	1.10 (1.06, 1.15)	<0.001
Q1	1044	1.00 (reference)		1.00 (reference)		1.00 (reference)		1.00 (reference)	
Q2	1043	1.25 (1.02, 1.52)	0.034	1.27 (1.03, 1.55)	0.022	1.27 (1.03, 1.56)	0.023	1.16 (0.95, 1.43)	0.151
Q3	1044	1.50 (1.23, 1.83)	<0.001	1.52 (1.25, 1.86)	<0.001	1.49 (1.22, 1.83)	<0.001	1.33 (1.08, 1.63)	0.007
Q4	1042	1.93 (1.59, 2.34)	<0.001	2.00 (1.64, 2.43)	<0.001	1.98 (1.62, 2.41)	<0.001	1.70 (1.38, 2.08)	<0.001
Ptrend			<0.001		<0.001		<0.001		<0.001
METS_IR									
per SD change		1.08 (1.07, 1.10)	<0.001	1.08 (1.07, 1.10)	<0.001	1.08 (1.07, 1.10)	<0.001	1.17 (1.15, 1.20)	<0.001
Q1	1045	1.00 (reference)		1.00 (reference)		1.00 (reference)		1.00 (reference)	
Q2	1044	1.56 (1.26, 1.95)	<0.001	1.61 (1.29, 2.01)	<0.001	1.60 (1.28, 1.99)	<0.001	2.07 (1.61, 2.67)	<0.001
Q3	1042	2.18 (1.77, 2.69)	<0.001	2.27 (1.84, 2.80)	<0.001	2.24 (1.81, 2.78)	<0.001	3.40 (2.56, 4.52)	<0.001
Q4	1042	3.57 (2.91, 4.38)	<0.001	3.65 (2.95, 4.51)	<0.001	3.59 (2.90, 4.45)	<0.001	6.79 (4.85, 9.52)	<0.001
Ptrend			<0.001		<0.001		<0.001		<0.001
TyG									
per SD change		1.99 (1.76, 2.24)	<0.001	2.06 (1.83, 2.33)	<0.001	2.06 (1.83, 2.33)	<0.001	1.87 (1.65, 2.12)	<0.001
Q1	1047	1.00 (reference)		1.00 (reference)		1.00 (reference)		1.00 (reference)	
Q2	1056	1.62 (1.32, 2.00)	<0.001	1.63 (1.32, 2.01)	<0.001	1.62 (1.31, 2.00)	<0.001	1.51 (1.21, 1.89)	<0.001
Q3	1039	1.87 (1.53, 2.30)	<0.001	1.95 (1.59, 2.39)	<0.001	1.94 (1.58, 2.38)	<0.001	1.73 (1.38, 2.15)	<0.001
Q4	1031	2.96 (2.41, 3.63)	<0.001	3.12 (2.53, 3.86)	<0.001	3.09 (2.50, 3.82)	<0.001	2.62 (2.10, 3.27)	<0.001
Ptrend			<0.001		<0.001		<0.001		<0.001
AIP									
per SD change		2.30 (1.80, 2.95)	<0.001	2.40 (1.87, 3.07)	<0.001	2.36 (1.84, 3.03)	<0.001	1.94 (1.49, 2.51)	<0.001
Q1	1093	1.00 (reference)		1.00 (reference)		1.00 (reference)		1.00 (reference)	
Q2	1012	1.28 (1.05, 1.56)	0.015	1.30 (1.06, 1.59)	0.012	1.29 (1.06, 1.58)	0.013	1.19 (0.97, 1.46)	0.098
Q3	1070	1.52 (1.25, 1.86)	<0.001	1.54 (1.26, 1.89)	<0.001	1.51 (1.24, 1.85)	<0.001	1.34 (1.09, 1.65)	0.005
Q4	998	1.93 (1.58, 2.35)	<0.001	1.99 (1.62, 2.43)	<0.001	1.97 (1.60, 2.40)	<0.001	1.69 (1.37, 2.08)	<0.001
Ptrend			<0.001		<0.001		<0.001		<0.001

Model 0: crude model.

Model 1: adjusted for Age, Gender.

Model 2: adjusted for Age, Gender, Smoking, Drinking, exercise.

Model 3: adjusted for Age, Gender, Smoking, Drinking, exercise, SBP, DBP, HR, BMI.

Quartile-based analyses showed that the ORs for all indices generally increased across ascending quartiles, and trend tests indicated significant dose-response relationships (P for trend< 0.05). In the fully adjusted model, the point estimates for CMM risk in the highest quartile were higher than those in the lowest quartile for all indices; except for RFM, which showed borderline significance, the differences for the remaining indices were statistically significant. Among these indices, METS-IR exhibited the largest risk gradient across quartiles, with an OR of 6.79 (95% CI 4.85-9.52) for the fourth versus the first quartile.

**Figure 2:**
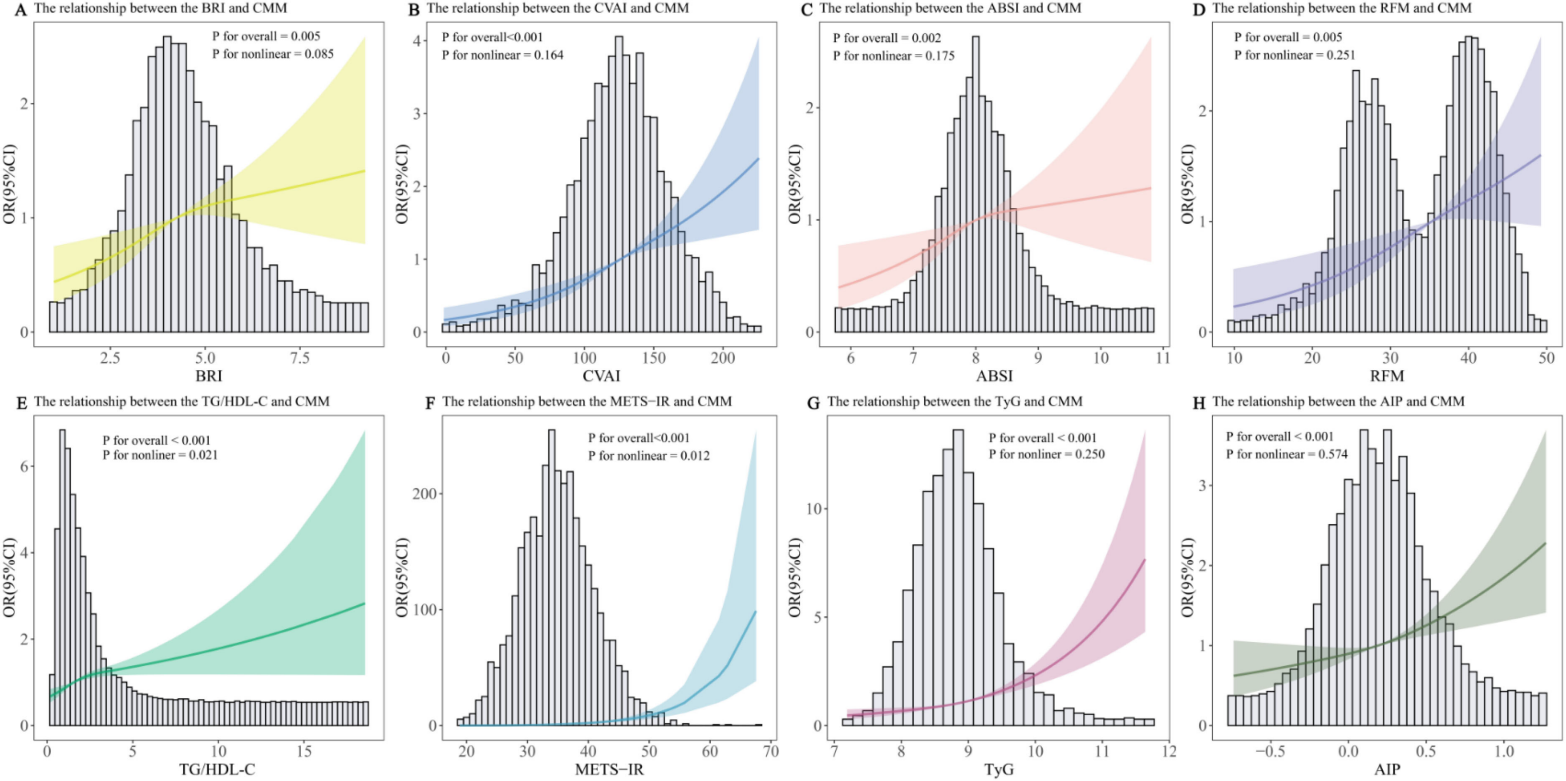
Restricted cubic spline of the relationship of CMM with BRI **(A)**, CVAI **(B)**, ABSI **(C)**, RFM **(D)**, TG/HDL-C **(E)**, METS-IR **(F)**, TyG **(G)**, AIP **(H)**. Potential nonlinear associations were assessed using restricted cubic spline models, with odds ratios (ORs) derived from logistic regression analyses. The ORs were adjusted for age, gender, smoking status, drinking status, exercise, SBP, DBP, HR, and BMI.

To further investigate the potential dose-response relationship, we applied a RCS model with three nodes to explore the nonlinear association between various indicators and CMM risk (**Figure 2**). BRI (P for overall = 0.005), CVAI (P for overall< 0.001), ABSI (P for overall = 0.002), RFM (P for overall = 0.005), TG/HDL-C (P for overall< 0.001), METS-IR (P for overall< 0.001), TyG (P for overall< 0.001), and AIP (P for overall< 0.001) were all positively associated with CMM. Tests for nonlinearity suggested largely linear relationships for BRI, CVAI, ABSI, RFM, TyG and AIP (all P for nonlinear > 0.05). In contrast, TG/HDL-C (P for nonlinear = 0.021) and METS-IR (P for nonlinear = 0.012) showed significant nonlinear positive associations, with steeper increases in CMM risk at higher levels.

### Discriminatory performance of indices for identifying CMM

**Figure 3:**
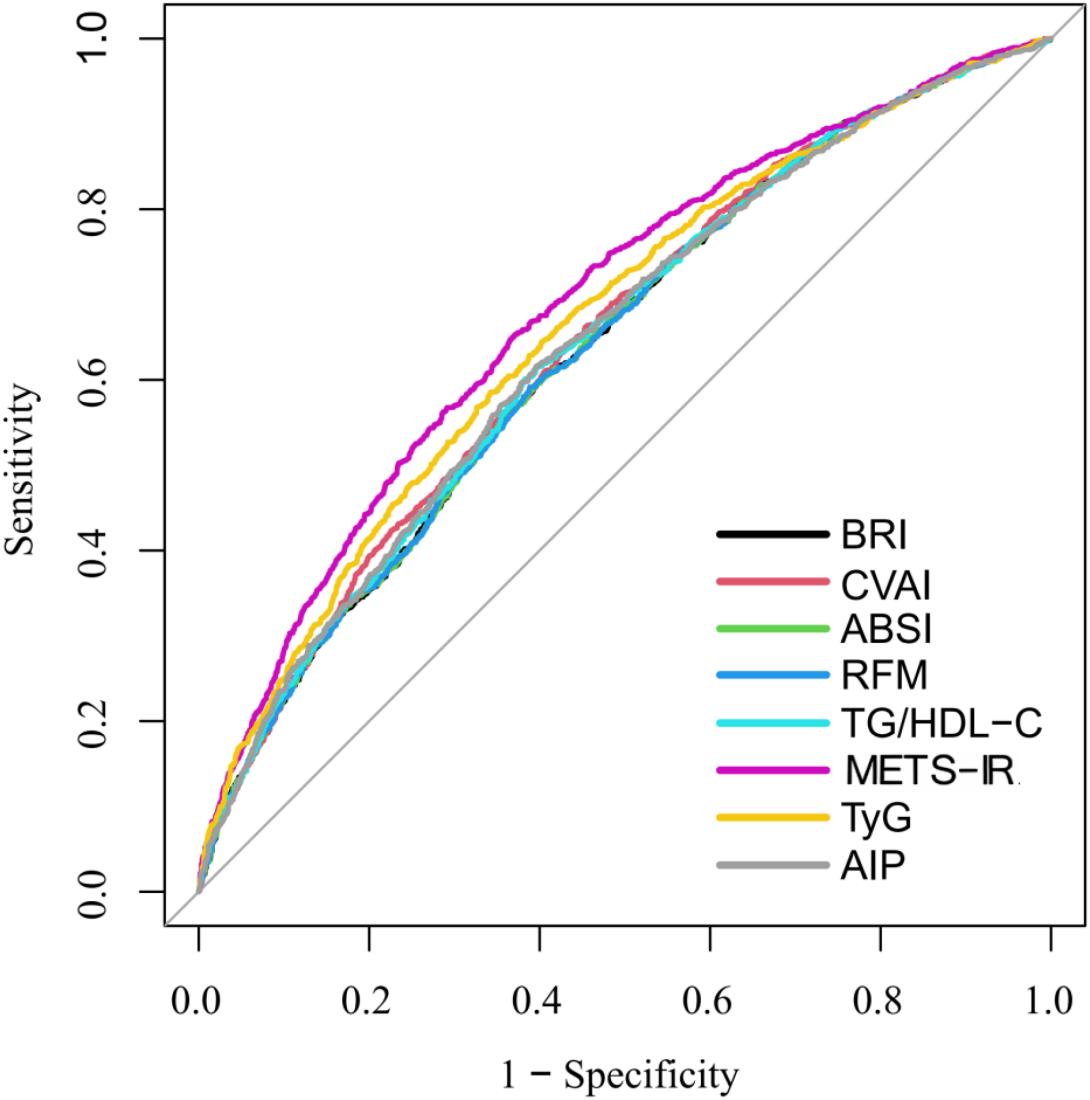
ROC curves of eight indices for the identification of CMM.

The discriminatory performance of the eight indices for identifying CMM was evaluated through ROC curve analysis, with the corresponding ROC curves shown in **Figure 3** and the adjusted AUC values summarized in **Table 3**.

**Table 3:** Predictive performance of different indicators for CMM.

Indicators	AUC(95%CI)	Adjusted AUC(95%CI)	P for comparison
BRI	0.587(0.568-0.606)	0.638(0.619-0.656)	Reference
CVAI	0.611(0.592-0.629)	0.646(0.628-0.665)	0.008
ABSI	0.534(0.515-0.554)	0.638(0.619-0.656)	0.687
RFM	0.552(0.532-0.571)	0.638(0.619-0.656)	0.961
TG/HDL-C	0.568(0.548-0.587)	0.642(0.624-0.661)	0.176
METS-IR	0.632(0.613-0.651)	0.685(0.667-0.703)	<0.001
TyG	0.611(0.592-0.630)	0.664(0.645-0.682)	<0.001
AIP	0.567(0.548-0.587)	0.644(0.625-0.662)	0.132

Adjusted for age, gender, smoking, drinking, exercise, SBP, DBP, HR, and BMI.

In the unadjusted models, METS-IR showed the highest discriminative ability (AUC = 0.632, 95% CI: 0.613-0.651), followed by TyG and CVAI (both AUC = 0.611), whereas ABSI had the lowest AUC (0.536, 95% CI: 0.516-0.555). After adjusting for confounding factors, the AUCs increased for all indicators. METS-IR remained the strongest predictor with an adjusted AUC of 0.685 (95% CI: 0.667-0.703), followed by the TyG (adjusted AUC = 0.664, 95% CI: 0.645-0.682). Overall, endocrine-metabolic indices demonstrated relatively higher discriminatory performance for CMM than obesity-related indices, although partial overlap in AUC values was observed between the two categories.

In pairwise comparisons using BRI as the reference, METS-IR (P< 0.001), TyG (P< 0.001), and CVAI (P = 0.008) showed stronger discriminative power, whereas the differences for ABSI, RFM, TG/HDL-C, and AIP were not statistically significant (all P > 0.05).

### Subgroup, interaction, and sensitivity analyses

**Figure 4:**
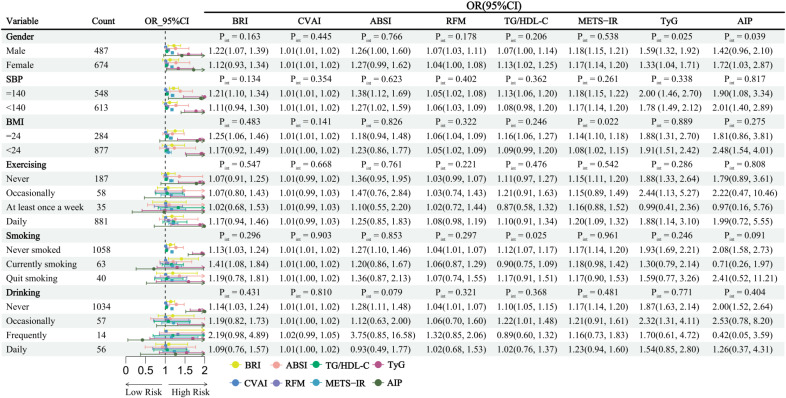
Stratified analyses of the associations between various indicators and CMM.

Subgroup analyses examining the associations between each index and CMM are presented in **Figure 4**. Interaction tests showed that sex significantly modified the associations of TyG (*P* for interaction = 0.025) and AIP (P for interaction = 0.039) with CMM. Specifically, TyG was more strongly associated with CMM in male (OR = 1.59, 95% CI: 1.32-1.92) than in female (OR = 1.33, 95% CI: 1.04-1.71), whereas AIP was significantly associated with CMM only in female (OR = 1.72, 95% CI: 1.03-2.87).

BMI also has a significant modifying effect on the relationship between METS-IR and CMM (P for interaction = 0.022), with a stronger association observed among participants with BMI ≥ 24 kg/m² (OR = 1.14, 95% CI: 1.10-1.18). After stratification by exercise, smoking, and drinking status, only TG/HDL-C showed a significant interaction with smoking status (P for interaction = 0.025), while the interactions of the other indicators were not significant (P for interaction > 0.05), indicating that the overall results were relatively robust.

In the sensitivity analysis (**Supplementary Table 1**), additional adjusted for TC and LDL-C did not materially change the results. The positive associations between all indices and CMM remained, and significant dose-response trends across quartiles persisted remained evident (all P for trend< 0.05). Specifically, for most indices, participants in Q4 had a higher risk of CMM than those in Q1. Although quartile-specific estimates for RFM did not consistently reach statistical significance, the overall trend for RFM remained significant.

## Discussion

To the best of our knowledge, few previous studies have systematically compared endocrine-metabolic indices and obesity-related indices in an elderly population to evaluate their ability to identify CMM. This study found that all examined indices were independently and positively associated with the presence of CMM after adjustment for potential confounders, and clear dose-response relationships were observed across indices. In addition, restricted cubic spline analyses suggested largely linear associations for most indices, while TG/HDL-C and METS-IR exhibited nonlinear relationships with a more pronounced increase in CMM risk at higher levels. Overall, endocrine-metabolic indices demonstrated relatively stronger associations and better discriminatory performance for CMM than obesity-related indices, although partial overlap in performance was observed between the two categories. These findings suggest that indicators reflecting endocrine-metabolic dysfunction may be more informative for identifying older individuals at increased risk of CMM.

Although the exact mechanisms linking obesity-related and endocrine-metabolic indices to CMM remain unclear (35), several plausible hypotheses lend support to our findings (36–40). Endocrine-metabolic indices largely reflect functional disturbances in insulin sensitivity, lipid metabolism, and vascular homeostasis, which are central to the pathogenesis of cardiometabolic disease (41). Experimental and clinical evidence indicates that insulin signaling promotes endothelial nitric oxide (NO) production, whereas IR induces oxidative stress and pro-inflammatory responses, leading to endothelial dysfunction and atherogenesis (36). In contrast, obesity-related indices primarily capture excess adiposity and its distribution, representing an upstream manifestation of chronic energy imbalance (42). Although adipose tissue expansion contributes to systemic inflammation, dyslipidemia, and metabolic dysfunction, these adverse effects often occur indirectly and may evolve over a longer time course before progressing to overt cardiometabolic disease (38). Importantly, endocrine-metabolic abnormalities may precede the development of overt obesity and Cardiovascular Disease (CVD). Previous studies have shown that IR-related markers, such as the TyG index, are more strongly associated with cardiovascular outcomes among individuals with normal weight (43, 44), suggesting that metabolic dysfunction may serve as an earlier and more sensitive signal of multimorbidity clustering (39). From a therapeutic perspective, improving endocrine-metabolic function may directly mitigate cardiometabolic risk (45), whereas effective management of obesity often depends on correcting underlying metabolic abnormalities (46, 47). For example, glucagon-like peptide-1 receptor agonists not only improve glycemic control but also reduce atherosclerotic cardiovascular risk through anti-inflammatory, antioxidative, and endothelial-protective mechanisms (40). Consistent with this concept, accumulating evidence suggests that individuals with metabolically unhealthy obesity experience substantially higher cardiovascular risk than their metabolically healthy counterparts despite similar body mass indices (48). Together, these findings support the notion that obesity-related indices primarily reflect risk burden, whereas endocrine-metabolic indices more directly capture the key pathological processes driving the development of CMM.

Notably, accumulating evidence supports the link between insulin resistance surrogates and early mechanical vascular impairment. A recent study demonstrated that the TG/HDL-C ratio was independently associated with arterial stiffness, whereas the TyG index showed no significant association in the same population, suggesting heterogeneity among metabolic markers in reflecting early vascular damage (49). The sensitivity of TG/HDL-C may stem from its direct reflection of atherogenic dyslipidemia, which promotes endothelial dysfunction and structural remodeling of the vessel wall (50, 51). However, in our study of older adults, METS-IR and TyG exhibited superior discriminatory performance for CMM compared to TG/HDL-C. This discrepancy may reflect different stages of the cardiovascular continuum, while TG/HDL-C excels at capturing early vascular stiffness and local arterial remodeling. Comprehensive markers like METS-IR and TyG, which integrate glucose and lipid metabolism, may more accurately reflect the systemic endocrine regulatory imbalance required to identify the clustering of multiple cardiometabolic disorders in aging populations (52).

Among endocrine-metabolic indices, IR-related markers appeared to play a particularly important role in identifying CMM. In the present study, METS-IR showed the strongest association with CMM and the highest discriminatory performance among all examined indices, suggesting that IR may represent a central pathological feature underlying the clustering of CMM in older adults (53). This finding is consistent with previous evidence showing that METS-IR is a robust indicator for predicting diabetes, CVD, and stroke, and may outperform several traditional metabolic markers in risk stratification (54–56). A large prospective cohort study conducted in China further demonstrated that individuals with elevated METS-IR had a substantially higher risk of incident cardiometabolic disease, supporting its relevance in long-term cardiometabolic risk assessment (57). Moreover, METS-IR has also been reported to be associated with increased all-cause mortality among patients with established CVD, highlighting its broader prognostic value (58). Taken together, these findings support the notion that endocrine-metabolic indices capturing IR as exemplified by METS-IR, may provide particularly informative signals for identifying individuals at high risk of CMM.

With respect to obesity-related indices, our findings indicate that these measures were also independently associated with cardiometabolic multimorbidity and exhibited clear dose-response relationships, highlighting the role of lipid accumulation and fat distribution in the development of CMM (59). Among these indices, CVAI showed relatively better discriminatory performance compared with other obesity indices, suggesting its potential utility for CMM risk assessment in older populations (31). This may be partly attributable to the fact that CVAI incorporates age- and lipid-related metabolic components (17), and was specifically developed for Chinese populations, thereby capturing both adiposity and metabolic characteristics more comprehensively (60–62). Previous studies have shown that each one-standard deviation increase in CVAI is associated with a 17% higher risk of incident stroke (62), and that CVAI outperforms traditional obesity indicators in predicting hypertension among Chinese adults (63). Moreover, a recent cross-sectional study reported that CVAI demonstrated better discriminatory ability for CMM than conventional obesity indices (38). Collectively, these findings support the relevance of obesity-related indices, particularly CVAI, in characterizing CMM risk, while also highlighting their complementary role relative to endocrine-metabolic indicators.

Further analyses using RCS models and subgroup stratification provided additional support for the robustness of our findings. Most indices exhibited approximately linear positive associations with CMM, whereas TG/HDL-C and METS-IR showed significant nonlinear relationships, characterized by a disproportionately steeper increase in CMM risk at higher levels. This pattern suggests that metabolic dysregulation may accumulate gradually at lower levels but accelerate once a critical threshold is exceeded, a finding consistent with previous observations in cardiometabolic research (27, 28). This nonlinear pattern may indicate a transition from compensated to decompensated metabolic states, in which cardiometabolic risk accumulates more rapidly (64). Subgroup analyses further indicated that the overall associations between the examined indices and CMM were largely consistent across major demographic and lifestyle strata. However, significant effect modification was observed for several indicators. Specifically, the association between TyG and CMM appeared stronger in men than in women, while AIP was significantly associated with CMM only among women. This sex-specific pattern may be related to differences in lipid metabolism, fat distribution, and hormonal regulation between men and women, which have been shown to influence cardiometabolic risk profiles (65, 66). In addition, BMI modified the association between METS-IR and CMM, with a more pronounced relationship observed in individuals with higher BMI levels. This may reflect greater adiposity-related metabolic dysregulation at higher BMI levels, which exacerbates IR and amplifies the metabolic abnormalities captured by METS-IR (67). A significant interaction was also detected between TG/HDL-C and smoking status, which may be partly explained by smoking-related alterations in lipid metabolism, including reduced high-density lipoprotein cholesterol and elevated triglyceride levels (37). Overall, these findings suggest the presence of population-specific heterogeneity, while supporting the general stability of the observed associations.

This study has several strengths that merit consideration. First, we systematically compared obesity indices and emerging endocrine-metabolic indices within the same elderly population, providing a comprehensive evaluation of their relative performance in identifying cardiometabolic multimorbidity. Second, the large community-based sample and extensive adjustment for demographic, lifestyle, and clinical covariates enhanced the statistical power and robustness of the findings. Third, by integrating multiple analytical approaches, including logistic regression, dose-response analyses, restricted cubic spline models, ROC analysis, subgroup analyses, and sensitivity analyses, we were able to assess the associations from complementary perspectives and verify the consistency of the results. Several limitations should also be acknowledged. First, the cross-sectional design precludes inference of temporal or causal relationships between the examined indices and cardiometabolic multimorbidity, and prospective studies are needed to confirm these findings. Second, some disease diagnoses and lifestyle variables were based on self-reported information, which may introduce recall or reporting bias. Third, although a wide range of potential confounders was adjusted for, residual confounding cannot be entirely excluded. Finally, the study population was derived from a single geographic region, which may limit the generalizability of the results to other populations with different genetic backgrounds, lifestyles, or metabolic profiles.

## Conclusion

In conclusion, both endocrine-metabolic indices and obesity indices were independently associated with cardiometabolic multimorbidity in older adults and demonstrated clear dose-response relationships. Overall, endocrine-metabolic indices showed relatively stronger associations and better discriminatory performance for identifying CMM than obesity indices. Among the examined indicators, endocrine-metabolic indices, particularly METS-IR, exhibited superior performance, while CVAI showed comparatively better discriminatory ability among obesity indices. These findings suggest that indices reflecting endocrine-metabolic dysfunction may be more informative for identifying older individuals at elevated risk of cardiometabolic multimorbidity in community settings. Integrating such indices into routine health assessments may help improve early risk stratification and targeted prevention strategies. Nevertheless, longitudinal studies are needed to confirm the predictive value of these indices and to determine their optimal thresholds for clinical and public health applications.

## Data Availability

The raw data supporting the conclusions of this article will be made available by the authors, without undue reservation.

## Glossary

CMM: Cardiometabolic multimorbidity
CVD: Cardiovascular Disease
CMD: cardiovascular metabolic disease
BRI: Body Roundness Index
CVAI: Chinese Visceral Adiposity Index
ABSI: A Body Shape Index
RFM: Relative Fat Mass
TG/HDL-C: Triglyceride/High-Density-Cholesterol ratio
METS-IR: Metabolic Score for Insulin Resistance
TyG: Triglyceride-Glucose index
AIP: Atherogenic Index of Plasma
RCS: restricted cubic spline
ROC: receiver operating characteristic
AUC: area under the curve
IR: insulin resistance
WC: waist circumference
BMI: body mass index
TG: triglycerides
HDL-C: high-density lipoprotein cholesterol
FPG: fasting plasma glucose
HR: heart rate
ALT: serum alanine aminotransferase
AST: aspartate aminotransferase
TBIL: total bilirubin
SCr: serum creatinine
BUN: blood urea nitrogen
TC: total cholesterol
LDL-C: low-density lipoprotein cholesterol
SBP: Systolic blood pressure
DBP: diastolic blood pressure
SD: standard deviation
IQR: interquartile range
OR: odds ratio
CI: confidence interval
NO: nitric oxide
