# Supplementary material for: Comparative discriminatory performance of emerging endocrine-metabolic indices versus obesity indices for cardiometabolic multimorbidity in older adults: a cross-sectional study
Source: Front Endocrinol (Lausanne). 2026 May 20;17:1776998. doi: 10.3389/fendo.2026.1776998 (PMC13229706; doi:10.3389/fendo.2026.1776998)
Supplement: Supplementary file 1 [file Table1.docx]

**Table S1** Associations of Different Indicators With CMM Risk After Additional Adjustment for TC and LDL-C

|  | Q1 | Q2 | Q3 | Q4 | P_trend_ |
| --- | --- | --- | --- | --- | --- |
| BRI |  |  |  |  | 0.008 |
| OR (95% CI) | 1.00 (reference) | 1.20 (0.96，1.50) | 1.26 (0.99，1.61) | 1.51 (1.13, 2.03) |  |
| CVAI |  |  |  |  | <0.001 |
| OR (95% CI) | 1.00 (reference) | 1.48 (1.17, 1.87) | 1.70 (1.31, 2.21) | 2.64 (1.91, 3.65) |  |
| ABSI |  |  |  |  | <0.001 |
| OR (95% CI) | 1.00 (reference) | 1.09 (0.88–1.34) | 1.39 (1.13–1.71) | 1.34 (1.09–1.66) |  |
| RFM |  |  |  |  | 0.041 |
| OR (95% CI) | 1.00 (reference) | 1.26 (0.99, 1.62) | 1.32 (0.86, 2.03) | 1.59 (0.97, 2.58) |  |
| TG/HDL-C |  |  |  |  | <0.001 |
| OR (95% CI) | 1.00 (reference) | 1.29 (1.03, 1.60) | 1.52 (1.22, 1.90) | 2.02 (1.63, 2.50) |  |
| METS-IR |  |  |  |  | <0.001 |
| OR (95% CI) | 1.00 (reference) | 2.01 (1.55, 2.59) | 3.23 (2.42, 4.31) | 6.42 (4.47, 9.21) |  |
| TyG |  |  |  |  | <0.001 |
| OR (95% CI) | 1.00 (reference) | 1.74 (1.39, 2.17) | 2.17 (1.73, 2.72) | 3.80 (3.03, 4.77) |  |
| AIP |  |  |  |  | <0.001 |
| OR (95% CI) | 1.00 (reference) | 1.31 (1.05, 1.62) | 1.56 (1.26, 1.94) | 2.01 (1.62, 2.48) |  |

Adjusted for age, gender, smoking, drinking, exercise, SBP, DBP, HR, BMI, TC and LDL-C.
